# Exposure-Response Association Between Concurrent Opioid and Benzodiazepine Use and Risk of Opioid-Related Overdose in Medicare Part D Beneficiaries

**DOI:** 10.1001/jamanetworkopen.2018.0919

**Published:** 2018-06-22

**Authors:** Inmaculada Hernandez, Meiqi He, Maria M. Brooks, Yuting Zhang

**Affiliations:** 1 Department of Pharmacy and Therapeutics, School of Pharmacy, University of Pittsburgh, Pittsburgh, Pennsylvania; 2 Department of Epidemiology, Graduate School of Public Health, University of Pittsburgh, Pittsburgh, Pennsylvania; 3 Department of Health Policy and Management, Graduate School of Public Health, University of Pittsburgh, Pittsburgh, Pennsylvania

## Abstract

**Importance:**

Concurrent benzodiazepine use is associated with an increased risk of opioid-related overdose; however, it remains unknown how the overdose risk varies with the days of exposure to both medications.

**Objective:**

To evaluate the exposure-response association between the days with concurrent prescription opioid and benzodiazepine use and the risk of overdose.

**Design, Setting, and Participants:**

Retrospective cohort study of Medicare Part D claims data from January 1, 2013, to December 31, 2014. Analyses were conducted in fall 2017 and spring 2018. Participants were Medicare Part D beneficiaries who filled at least 1 prescription for an opioid in 2014.

**Exposures:**

Patients were divided into 2 groups based on whether they had opioid and benzodiazepine supplies on the day before overdose or censoring. The first group only had a supply of opioids (n = 50 583) and the second group had supplies of opioids and benzodiazepines (n = 20 665). The second group was further categorized into 4 subgroups based on the cumulative number of days with overlapping supplies of opioids and benzodiazepines: 1 to 90 days (n = 3603), 91 to 180 days (n = 2930), 181 to 270 days (n = 4082), and 271 days or more (n = 10 050).

**Main Outcomes and Measures:**

Cox proportional hazard models were constructed to compare opioid-related overdose (including fatal and nonfatal overdoses) across time-dependent treatment groups, controlling for demographic characteristics, insurance factors, clinical characteristics, and number of unique opioid and benzodiazepine prescribers.

**Results:**

Of 71 248 total participants, 25 600 (35.9%) were male and 59 532 (83.6%) were white. Mean (SD) age was 66.5 (14.8) years. On the day before overdose or censoring event, 20 665 of 71 248 patients with an opioid prescription (29.0%) were concurrently using benzodiazepines and 14 132 of 20 665 concurrent users (68.4%) had more than 180 days of overlapping supplies of both medications. The risk of overdose was highest on the first days of concurrent opioid and benzodiazepine use and decreased over time; compared with opioid use alone, the hazard ratio for overdose was 5.05 (95% CI, 3.68-6.93) during the first 90 days of concurrent opioid and benzodiazepine use and 1.87 (95% CI, 1.25-2.80) for days 91 to 180 among those who did not have an event before 90 days.

**Conclusions and Relevance:**

During the first 90 days, concurrent benzodiazepine use is associated with a 5-fold increase in the risk of opioid-related overdose. The implementation of policies deterring concurrent opioid and benzodiazepine use is warranted. Patients using both medications should be closely monitored, particularly during the first days of concurrent use.

## Key Points

**Question:**

How does the risk of overdose change with the number of days with concurrent opioid and benzodiazepine use?

**Findings:**

In this cohort study of US Medicare data, the overdose risk associated with concurrent benzodiazepine use changed over time. Concurrent benzodiazepine use was associated with a 5-fold increase in the risk of opioid-related overdose during the initial 90 days and an increase of 1.87 times on days 91 to 180.

**Meaning:**

Policy interventions should focus on preventing concurrent opioid and benzodiazepine use in the first place instead of reducing the length of concurrent use. Patients using both medications should be closely monitored, particularly during the first days of concurrent use.

## Teaser

This cohort study investigates the associations between concurrent prescription opioid and benzodiazepine use and the risk of opioid overdose among Medicare Part D beneficiaries in 2013 and 2014.

## Introduction

Between 1999 and 2015, opioid-related overdoses nearly tripled in the United States, and in 2015 alone more than 33 000 deaths were due to opioid-related overdoses.^1,2^ It is estimated that 30% of fatal opioid-related overdoses involve the concurrent use of benzodiazepines.^3^ The concurrent administration of benzodiazepines increases the risk of opioid-related overdose because of the combination of their depressant effects on the central nervous system’s controls for respiration.^4^ For this reason, the Centers for Disease Control and Prevention recommends against their concurrent use.^4^ Despite this recommendation, the concurrent use of opioids and benzodiazepines has increased by more than 40% in the last 12 years,^5^ and in 2013 more than 20% of patients taking opioids used benzodiazepines concurrently.^6,7^

To our knowledge, 2 studies have evaluated the association between concurrent opioid and benzodiazepine use and overdose. Specifically, Park et al^7^ used claims from the Veterans Health Administration and found that concurrent opioid and benzodiazepine use was associated with a 3-fold increase in the risk of fatal overdose. Sun et al^6^ analyzed claims from a commercially insured population to note that concurrent use was associated with 2.15 times greater odds of an emergency department visit or inpatient admission for overdose.^7^ Both studies adjusted for a comprehensive list of patient characteristics but did not control for the number of opioid prescribers, which has been shown to increase the risk of overdose.^8,9,10,11^ Moreover, these previous investigations did not evaluate how the risk of opioid-related overdose changes with the duration of concurrent opioid and benzodiazepine use. A recent report from the Centers for Medicare and Medicaid Services (CMS) showed that 33.3% of Medicare Part D patients who did not have cancer and were not in hospice filled prescriptions for opioids in 2015 and, among these patients, 24.0% had concurrently filled a prescription for benzodiazepines.^12^ Despite the prevalence of concurrent opioid and benzodiazepine use in Medicare, no study to our knowledge has examined the association between concurrent opioid and benzodiazepine use and overdose in the Medicare population.

Our study fills these gaps in the literature. We used Medicare claims data from January 1, 2013, to December 31, 2014, to evaluate the association between concurrent use of prescription opioids and benzodiazepines and the risk of opioid-related overdose (including fatal and nonfatal overdoses) while controlling for a comprehensive list of patient demographic characteristics, insurance, clinical characteristics, and, more interestingly, the number of unique clinicians who prescribed opioids or benzodiazepines for each patient. We further examined the exposure-response association between the number of days with overlapping supply of prescription opioids and benzodiazepines and the risk of opioid-related overdose.

## Methods

### Data Source and Sample Selection

**Figure 1.**
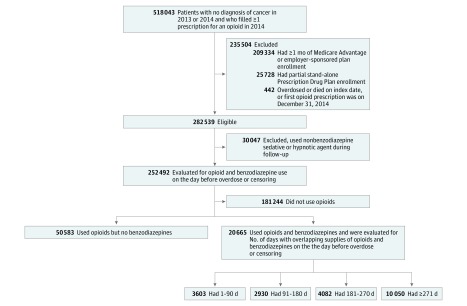
Selection of the Study Sample Patients were classified into treatment groups based on whether they used opioids only or opioids and benzodiazepines on the day before overdose or censoring event. Patients who used opioids and benzodiazepines were further classified according to the cumulative number of days with overlapping supplies of opioids and benzodiazepines as of the day before overdose or censoring.

We obtained 2013 to 2014 claims data from a 5% random sample of Medicare Part D beneficiaries from CMS. Guided by the methods used by CMS to identify concurrent opioid and benzodiazepine use, we extracted our study cohort in the following steps.^12^ First, we identified patients who did not have cancer and who filled at least 1 prescription for opioids^13,14,15^ (Table 1) in 2014 (Figure 1).^6^ Index day was defined as the day of the first prescription for an opioid in 2014. Second, we excluded patients who were enrolled in Medicare Advantage prescription drug plans or employer-sponsored plans because we did not have access to their medical claims. We also excluded beneficiaries who had partial enrollment in a stand-alone Medicare Part D Prescription Drug Plan. Patients who died in 2014 but were continuously enrolled in a stand-alone Prescription Drug Plan until the month of death were included in our sample. We followed all beneficiaries from the index date until the first of the following events: overdose, death, or end of the study period (December 31, 2014). Patients who overdosed or died on the index date, or whose first prescription for an opioid was on December 31, 2014, were excluded. The University of Pittsburgh institutional review board declared this study exempt and did not require patient informed consent because deidentified data were used. Strengthening the Reporting of Observational Studies in Epidemiology (STROBE) reporting guidelines were followed in the analysis and reporting of this study.

**Table 1.** Baseline Patient Characteristics by Time-Dependent Treatment Assignment^a^

Characteristic	Opioid Use and No Benzodiazepine Use (n = 50 583)	1-90 d With Concurrent Opioid and Benzodiazepine Use (n = 3603)	91-180 d With Concurrent Opioid and Benzodiazepine Use (n = 2930)	181-270 d With Concurrent Opioid and Benzodiazepine Use (n = 4082)	≥271 d With Concurrent Opioid and Benzodiazepine Use (n = 10 050)
Demographic characteristics					
Age, mean (SD), y	67.65 (14.72)	70.60 (16.05)	66.23 (14.00)	63.53 (14.30)	60.51 (13.10)
Male, No. (%)	18 948 (37.46)	1024 (28.42)	875 (29.86)	1153 (28.25)	3600 (35.82)
Race, No. (%)					
White	41 255 (81.56)	3169 (87.95)	2577 (87.95)	3590 (87.95)	8941 (88.97)
Black	7060 (13.96)	284 (7.88)	269 (9.18)	334 (8.18)	823 (8.19)
Hispanic	824 (1.63)	71 (1.97)	41 (1.40)	66 (1.62)	102 (1.01)
Other	1444 (2.85)	79 (2.19)	43 (1.47)	92 (2.25)	184 (1.83)
Insurance characteristics, No. (%)					
Disability	19 374 (38.30)	1150 (31.92)	1249 (42.63)	2076 (50.86)	6301 (62.70)
Medicaid eligibility	25 300 (50.02)	1801 (49.99)	1495 (51.02)	2324 (56.93)	6551 (65.18)
Low-income subsidy	3706 (7.33)	223 (6.19)	233 (7.95)	337 (8.26)	938 (9.33)
Clinician characteristics, mean (SD)					
No. of unique opioid prescribers^b^	2.19 (1.55)	2.02 (1.48)	1.39 (1.82)	2.49 (1.92)	2.21 (1.51)
No. of unique benzodiazepine prescribers^b^	0.28 (0.64)	1.33 (0.69)	1.61 (0.96)	1.73 (1.10)	1.63 (0.96)
Clinical characteristics, No. (%)^c^					
Acquired hypothyroidism	13 532 (26.75)	1177 (32.67)	929 (31.71)	1264 (30.97)	2686 (26.73)
Acute myocardial infarction	2689 (5.32)	257 (7.13)	156 (5.32)	178 (4.36)	439 (4.37)
Alcohol use disorder^d^	48 (0.09)	5 (0.14)	2 (0.07)	2 (0.05)	3 (0.03)
Alzheimer disease or other dementia	7456 (14.74)	972 (26.98)	507 (17.30)	617 (15.12)	1103 (10.98)
Anemia	29 503 (58.33)	2363 (65.58)	1883 (64.27)	2461 (60.29)	5678 (56.50)
Anxiety^e^	1229 (2.43)	203 (5.63)	232 (7.92)	329 (8.06)	1067 (10.62)
Asthma	10 951 (21.65)	883 (24.51)	796 (27.17)	1149 (28.15)	2797 (27.83)
Atrial fibrillation	6719 (13.28)	652 (18.10)	365 (12.46)	433 (10.61)	742 (7.38)
Benign prostatic hyperplasia	5327 (10.53)	370 (10.27)	250 (8.53)	302 (7.40)	784 (7.80)
Bipolar disorder^f^	271 (0.54)	50 (1.39)	37 (1.26)	38 (0.93)	133 (1.32)
Cataract	24 284 (48.01)	2027 (56.26)	1390 (47.44)	1678 (41.11)	3430 (34.13)
Chronic kidney disease	14 690 (29.04)	1196 (33.19)	853 (29.11)	1098 (26.90)	2372 (23.60)
Chronic obstructive pulmonary disease	19 283 (38.12)	1547 (42.94)	1322 (45.12)	1896 (46.45)	4893 (48.69)
Depression	27 227 (53.83)	2480 (68.83)	2155 (73.55)	3092 (75.75)	7665 (76.27)
Diabetes	21 798 (43.09)	1539 (42.71)	1241 (42.35)	1670 (40.91)	3945 (39.25)
Drug use disorder^g^	15 (0.03)	0	0	1 (0.02)	0
Fibromyalgia^h^	1919 (3.79)	86 (2.39)	111 (3.79)	191 (4.68)	401 (3.99)
Glaucoma	8718 (17.24)	719 (19.96)	487 (16.62)	613 (15.02)	1197 (11.91)
Heart failure	16 320 (32.26)	1377 (38.22)	1016 (34.68)	1224 (29.99)	2789 (27.75)
Hip or pelvic fracture	2590 (5.12)	264 (7.33)	161 (5.49)	189 (4.63)	350 (3.48)
Hyperlipidemia	36 575 (72.31)	2694 (74.77)	2166 (73.92)	2878 (70.50)	6965 (69.30)
Hypertension	41 241 (81.53)	2972 (82.49)	2383 (81.33)	3250 (79.62)	7821 (77.82)
Ischemic heart disease	24 891 (49.21)	1998 (55.45)	1553 (53.00)	1978 (48.46)	4669 (46.46)
Osteoporosis	11 179 (22.10)	1012 (28.09)	706 (24.10)	936 (22.93)	1803 (17.94)
Pain^i^	38 554 (76.22)	2349 (65.20)	1908 (65.12)	2668 (65.36)	6453 (64.21)
Posttraumatic stress disorder^j^	44 (0.09)	10 (0.28)	6 (0.20)	12 (0.29)	33 (0.33)
Psychosis^k^	305 (0.60)	36 (1.00)	19 (0.65)	26 (0.64)	53 (0.53)
Rheumatoid arthritis or osteoarthritis	37 175 (73.49)	2610 (72.44)	2191 (74.78)	3084 (75.55)	7454 (74.17)
Schizophrenia^l^	133 (0.26)	22 (0.61)	10 (0.34)	10 (0.24)	36 (0.36)
Stroke or transient ischemic attack	8164 (16.14)	765 (21.23)	512 (17.47)	623 (15.26)	1371 (13.64)

^a^ Treatment assignment was defined based on the opioid and benzodiazepine use and the history of benzodiazepine use on the day before overdose or censoring.

^b^ The number of unique opioid prescribers and the number of unique benzodiazepine prescribers are clinicians who prescribed at least 1 prescription for each of these medications in 2014.

^c^ We used Centers for Medicare and Medicaid Services Chronic Condition Data Warehouse definitions of all clinical characteristics except for a recent history of alcohol use disorder, anxiety, bipolar disorder, drug use disorder, fibromyalgia, pain, posttraumatic stress disorder, psychosis, and schizophrenia, which are not Centers for Medicare and Medicaid Services priority conditions.^13^

^d^ Alcohol use disorder was defined as having at least 1 inpatient or outpatient claim with *International Classification of Diseases, Ninth Revision* (*ICD-9*) code 265.2, 291.1-291.3, 291.5-291.9, 303.0, 303.9, 305.0, 357.5, 425.5, 535.3, 571.0-571.3, 980.x, or V11.3 in the year before the index date.^6^

^e^ Anxiety was defined as having at least 1 inpatient or outpatient claim with *ICD-9* code 300.0 in the year before the index date.^14^

^f^ Bipolar disorder was defined as having at least 1 inpatient or outpatient claim with *ICD-9* code 296.0x, 296.1x, or 296.4x-296.9x in the year before the index date.^15^

^g^ Drug use disorder was defined as having at least 1 inpatient or outpatient claim with *ICD-9* code 292.x, 304.x, 305.2-305.9, or V65.42.6 in the year before the index date.

^h^ Fibromyalgia was defined as having at least 1 inpatient or outpatient claim with *ICD-9* code 729.1 in the year before the index date.^14^

^i^ Pain was defined as having at least 1 inpatient or outpatient claim with *ICD-9* code 719.4 (joint pain), 723.1 (cervicalgia), 724.1 (pain in thoracic spine), 724.2 (lumbago), 724.3 (sciatica), 724.5 (backache unspecified), 729.5 (limb pain), 784.0 (headache), 786.5 (chest pain), 789.0 (abdominal pain), 350.1 (trigeminal neuralgia), 350.2 (atypical face pain), 346 (migraine), 250.6 (diabetes with neurological manifestations), 307.8 (pain disorders related to psychological factors), 354.x (mononeuritis of upper limb), or 357.2 (polyneuropathy in diabetes) in the year before the index date.^14^

^j^ Posttraumatic stress disorder was defined as having at least 1 inpatient or outpatient claim with *ICD-9* code 309.81 in the year before the index date.^14^

^k^ Psychosis was defined as having at least 1 inpatient or outpatient claim with *ICD-9* code 293.8, 296.04, 296.14, 296.44, 296.54, 297.x, or 298.x in the year before the index date.^6^

^l^ Schizophrenia was defined as having at least 1 inpatient or outpatient claim with *ICD-9* code 295.x in the year before the index date.^15^

We collected the prescriptions filled by the remaining beneficiaries for benzodiazepines or for nonbenzodiazepine sedative or hypnotic agents (these are also central nervous system depressants but are less potent and have a shorter duration of action than benzodiazepines, so it is important to identify use of them separately) and excluded patients who filled at least 1 prescription for nonbenzodiazepine sedative or hypnotic agents. Then, using the dates when prescriptions were filled and the days of supply, we defined 3 time-dependent variables for each patient on each day between the index date and the first of the following events: overdose, death, or December 31, 2014. The first time-dependent variable indicated whether a patient had a supply of opioids on the given day, the second denoted whether a patient had a supply of both opioids and benzodiazepines on the given day, and the third indicated the cumulative number of days that a patient had overlapping supplies of both medications since the index date. Based on these time-dependent variables, we defined 6 mutually exclusive time-dependent treatment groups: patients who did not have a supply of opioids on the day before overdose or censoring, patients who had a supply of opioids but not of benzodiazepines on the day before overdose or censoring, and patients who had a supply of opioids and benzodiazepines on the day before overdose or censoring and a history of 1 to 90 days, 91 to 180 days, 181 to 270 days, and 271 days or more with overlapping supplies of both medications. We allowed for potential interruptions of concurrent opioid and benzodiazepine use in the calculation of the cumulative number of days with overlapping supplies of both medications. For instance, if a patient had 90 days with overlapping supplies of both medications, an interruption for 15 days, and then 30 additional days with concurrent use, including the day before the patient overdosed or was censored, the patient would be categorized into the treatment group with concurrent opioid and benzodiazepines use and a history of 91 to 180 days with overlapping supplies of both medications. We allowed for these interruptions because patients recently exposed to benzodiazepines would have likely developed some degree of tolerance to their effects from the prior exposure. If we had not allowed for interruptions in the calculation of the cumulative number of days with overlapping supplies of both medications, reexposures to concurrent opioid and benzodiazepine use would have been categorized in a similar manner as first-time exposures, which would not have been precise from a pharmacological perspective. We selected the 90-, 180-, and 270-day cutoffs to have time windows with a similar duration and facilitate the interpretation of results. Treatment groups were assigned based on the supply of opioids and benzodiazepines on the day before overdose or censoring for 2 reasons: first, given the rapid onset of the depressant effects of benzodiazepines, overdose events should only be attributed to the concurrent benzodiazepine use group when patients had a supply of both medications immediately before their occurrence, and second, categorizing patients into treatment groups based on the supply of both medication classes on the day of overdose would not have been adequate because it would not have been possible to determine whether patients were exposed to both medications before the occurrence of the overdose. Patients who did not use opioids on the day before overdose or censoring were not included in analyses. This is because the objective of the study was to evaluate how the risk of overdose associated with opioids increases with the concurrent use of benzodiazepines, so only patients using opioids on the day before overdose or censoring were included.

### Outcomes

The main outcome of interest was opioid-related overdose, which included fatal and nonfatal overdoses. Following previously published definitions, overdose was defined as having an inpatient or outpatient claim with *International Classification of Diseases, Ninth Revision* codes 965.0, 965.00, 965.02, 965.09, E850.1, E850.2, E950.0, E980.0, 965.1, or E850.0.5.^16^

### Covariates

In our analyses, we adjusted for patient-level demographic characteristics, health insurance factors, a comprehensive list of clinical characteristics, and the number of unique clinicians who prescribed opioids or benzodiazepines to each patient in 2014. Demographic characteristics included age, sex, and race. Health insurance factors included eligibility for Medicare due to disability, eligibility for Medicaid coverage, and low-income subsidy. Clinical characteristics included all CMS priority chronic conditions^13^ except those related to cancer, as well as the following variables: a recent history of alcohol use disorder, anxiety, bipolar disorder, drug use disorder, fibromyalgia, pain, posttraumatic stress disorder, psychosis, and schizophrenia (Table 1).

### Statistical Analysis

We compared baseline characteristics across treatment groups using analysis of variance for continuous variables and χ^2^ for categorical variables. We constructed Kaplan-Meier time-to-event curves to compare the unadjusted cumulative incidence rates of opioid-related overdose between patients who only had an opioid supply and those who had concurrent opioid and benzodiazepine supplies on the day before overdose or censoring. We constructed Cox proportional hazard models that included indicator variables for 4 time-dependent treatment groups (the opioid-only treatment group was used as the reference) and controlled for all covariates listed in the Covariates subsection and in Table 1. We also reported the hazard ratios (HRs) for each of the covariates for which we adjusted. Time 0 was the index date, and the time at risk was censored at death or the end of the study (December 31, 2014). The threshold for statistical significance was set at 2-sided *P *< .05. Analyses were conducted with SAS statistical software version 9.4 (SAS Institute) and were performed in fall 2017 (original analyses) and in spring 2018 (revision).

In sensitivity analyses, we constructed Cox proportional hazard models that controlled for the same covariates as mentioned previously except for numbers of unique opioid prescribers and unique benzodiazepine prescribers.

## Results

### Patient Characteristics and Prevalence of Concurrent Opioid and Benzodiazepine Use

Of 71 248 total participants, 25 600 (35.9%) were male and 59 532 (83.6%) were white. Mean (SD) age was 66.5 (14.8) years. Among Medicare Part D beneficiaries who used prescription opioids on the day before overdose or censoring, 20 665 of 71 248 (29.0%) were concurrently using benzodiazepines, and 14 132 of 20 665 concurrent users (68.4%) had overlapping supplies of both medications for more than 180 days (Figure 1).

White, disabled, dual-eligible beneficiaries and those eligible for low-income subsidy were more likely to use prescription opioids and benzodiazepines concurrently (Table 1). The numbers of opioid prescribers and benzodiazepine prescribers increased the likelihood of concurrent opioid and benzodiazepine use. Depression and anxiety were associated with higher concurrent opioid and benzodiazepine use and with more days with overlapping supplies of opioids and benzodiazepines.

### Association Between Concurrent Prescription Opioid and Benzodiazepine Use and Risk of Opioid-Related Overdose

#### Unadjusted Results

The frequency of opioid-related overdose was higher for patients using opioids and benzodiazepines concurrently (0.60%) than for patients using only opioids (0.33%) (Table 2 and eFigure in the Supplement). The frequency of overdose events was particularly high in the first days with concurrent opioid and benzodiazepine use but decreased over time: 1.64% for days 1 to 90 of concurrent use, 1.09% for days 91 to 180, 0.47% for days 181 to 270, and 0.14% for day 271 or later.

**Table 2.** Number of Opioid-Related Overdose Events by Opioid and Benzodiazepine Use on the Day Before Overdose or Censoring^a^

Treatment on the Day Before Overdose or Censoring	Events, No. (%)
Opioid use and no benzodiazepine use (n = 50 583)	166 (0.33)
Opioid and benzodiazepine use (n = 20 665)	124 (0.60)
1-90 d with concurrent opioid and benzodiazepine use (n = 3603)	59 (1.64)
91-180 d with concurrent opioid and benzodiazepine use (n = 2930)	32 (1.09)
181-270 d with concurrent opioid and benzodiazepine use (n = 4082)	19 (0.47)
≥271 d with concurrent opioid and benzodiazepine use (n = 10 050)	14 (0.14)

^a^ Treatment groups were defined in a time-dependent manner, based on the use of opioids and benzodiazepines and the history of benzodiazepine use on the day before overdose or censoring event (death or end of the study period).

#### Adjusted Results

**Figure 2.**
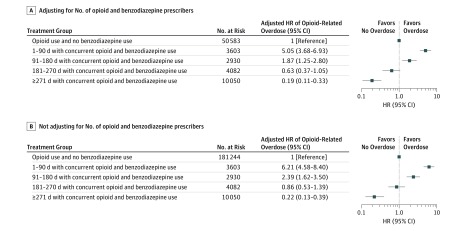
Adjusted Hazard Ratios (HRs) of Opioid-Related Overdose for Treatment Groups, Before and After Adjusting for Number of Opioid and Benzodiazepine Prescribers In both panels, adjusted HRs were estimated with Cox proportional hazard models that controlled for age, sex, race, eligibility for Medicare due to disability, eligibility for Medicaid coverage, low-income subsidy, all Centers for Medicare and Medicaid Services priority chronic conditions except those related to cancer, a recent history of alcohol use disorder, anxiety, bipolar disorder, drug use disorder, fibromyalgia, pain, posttraumatic stress disorder, psychosis, and schizophrenia. A, The Cox model also adjusted for the number of unique opioid prescribers and the number of unique benzodiazepine prescribers.

Figure 2 shows adjusted HRs for opioid-related overdose with different durations of concurrent opioid and benzodiazepine use relative to opioid use alone. The risk of overdose was highest during the first days of concurrent opioid and benzodiazepine use but then decreased over time: compared with opioid use alone, the HR for overdose was 5.05 (95% CI, 3.68-6.93) for days 1 to 90 of concurrent opioid and benzodiazepine use. Among those who were exposed for more than 90 days to concurrent use and did not overdose, the HR of overdose in days 91 to 180 was 1.87 (95% CI, 1.25-2.80) compared with opioid use alone. For those who did not overdose by day 180 of concurrent use, the hazards of overdose after day 181 of concurrent opioid and benzodiazepine use were not higher than with opioid use alone (HR, 0.63; 95% CI, 0.37-1.05 for days 181 to 270 and HR, 0.19; 95% CI, 0.11-0.33 for day 271 or later).

#### Results From Sensitivity Analyses

Adjusted HRs for opioid-related overdose considerably increased when Cox models were not adjusted for the numbers of opioid and benzodiazepine prescribers (Figure 2). Compared with opioid use alone, HRs for overdose were 6.21 (95% CI, 4.58-8.40) for days 1 to 90 of concurrent opioid and benzodiazepine use, 2.39 (95% CI, 1.62-3.50) for days 91 to 180, 0.86 (95% CI, 0.53-1.39) for days 181 to 270, and 0.22 (95% CI, 0.13-0.39) for day 271 or later.

### Association Between Other Covariates and Risk of Opioid-Related Overdose

The risk of overdose was lower for black patients than for white patients (HR, 0.52; 95% CI, 0.34-0.79) (Table 3). The risk of overdose increased by 7% (95% CI, 2%-12%) for every additional opioid prescriber, and by 19% (95% CI, 9%-31%) for every additional benzodiazepine prescriber. The risk of overdose also increased with certain conditions: chronic kidney disease (HR, 1.58; 95% CI, 1.21-2.08), chronic obstructive pulmonary disease (HR, 1.50; 95% CI, 1.15-1.95), depression (HR, 1.69; 95% CI, 1.23-2.34), and a history of stroke or transient ischemic attack (HR, 1.56; 95% CI, 1.15-2.01).

**Table 3.** Hazard Ratios of Opioid-Related Overdose Adjusted for Covariates

Variable	Hazard Ratio (95% CI)^a^
Treatment on the day before overdose or censoring^b^	
Opioid use and no benzodiazepine use	1 [Reference]
1-90 d with concurrent opioid and benzodiazepine use	5.05 (3.68-6.93)
91-180 d with concurrent opioid and benzodiazepine use	1.87 (1.25-2.80)
181-270 d with concurrent opioid and benzodiazepine use	0.63 (0.37-1.05)
≥271 d with concurrent opioid and benzodiazepine use	0.19 (0.11-0.33)
Demographic characteristics	
Age per y	0.97 (0.96-0.99)
Male vs female	0.94 (0.70-1.26)
Black vs white	0.52 (0.34-0.79)
Hispanic vs white	0.47 (0.15-1.47)
Other vs white	1.27 (0.67-2.40)
Insurance characteristics	
Disability	1.13 (0.74-1.71)
Medicaid eligibility	0.90 (0.67-1.22)
Low-income subsidy	0.98 (0.61-1.55)
Clinician characteristics	
No. of unique opioid prescribers	1.07 (1.02-1.12)
No. of unique benzodiazepine prescribers	1.19 (1.09-1.31)
Clinical characteristics	
Acquired hypothyroidism	0.82 (0.62-1.09)
Acute myocardial infarction	1.16 (0.72-1.87)
Alcohol use disorder^c^	NA
Alzheimer disease or other dementia	1.38 (0.97-1.96)
Anemia	1.40 (1.05-1.86)
Anxiety	1.21 (0.74-1.96)
Asthma	1.12 (0.86-1.47)
Atrial fibrillation	0.60 (0.38-0.93)
Benign prostatic hyperplasia	1.14 (0.73-1.78)
Bipolar disorder	1.77 (0.78-4.02)
Cataract	0.63 (0.47-0.86)
Chronic kidney disease	1.58 (1.21-2.08)
Chronic obstructive pulmonary disease	1.50 (1.15-1.95)
Depression	1.69 (1.23-2.34)
Diabetes	0.92 (0.70-1.20)
Drug use disorder^c^	NA
Fibromyalgia	0.94 (0.51-1.73)
Glaucoma	0.89 (0.62-1.28)
Heart failure	1.01 (0.75-1.36)
Hip or pelvic fracture	1.90 (1.18-3.03)
Hyperlipidemia	0.76 (0.56-1.02)
Hypertension	1.00 (0.70-1.43)
Ischemic heart disease	1.38 (1.03-1.86)
Osteoporosis	0.80 (0.57-1.11)
Pain	0.77 (0.60-0.99)
Posttraumatic stress disorder^c^	NA
Psychosis	0.53 (0.07-3.77)
Rheumatoid arthritis or osteoarthritis	1.42 (1.05-1.94)
Schizophrenia	2.40 (0.76-7.62)
Stroke or transient ischemic attack	1.56 (1.15-2.10)

Abbreviation: NA, not available.

^a^ Hazard ratios were estimated with Cox proportional hazard models that controlled for all the covariates listed in the table.

^b^ Treatment assignment was defined based on the opioid and benzodiazepine use and the history of benzodiazepine use on the day before the first of the following events: overdose, death, or end of the study (December 31, 2014).

^c^ Hazard ratios for alcohol use disorder, drug use disorder, and posttraumatic stress disorder could not be estimated because of insufficient events.

## Discussion

To our knowledge, this study is the first to use Medicare data to examine the association between the concurrent use of prescription opioids and benzodiazepines and the risk of opioid-related overdose. Our study yielded 3 main findings. First, we found that 29% of Medicare Part D beneficiaries who did not have cancer and who used prescription opioids concurrently filled prescriptions for benzodiazepines. Second, we found that the risk of opioid-related overdose is particularly high during the first days with concurrent opioid and benzodiazepine use and then decreases over time. Specifically, during the first 90 days of concurrent benzodiazepine use, the risk of opioid-related overdose is 5 times higher compared with opioid use alone. Third, the numbers of opioid and benzodiazepine prescribers were associated with an increased likelihood of concurrent opioid and benzodiazepine use and an increased risk of overdose and were strong confounders in examining the association between concurrent use and overdose.

In our analyses, we categorized participants into time-dependent treatment groups and modeled how the risk of overdose with concurrent opioid and benzodiazepine use compared with opioid use alone based on the cumulative duration of concurrent opioid and benzodiazepine use. This time-dependent method can complicate the interpretation of our results. Specifically, we found that during the first 90 days a patient is exposed concurrently to opioids and benzodiazepines, the risk of overdose is 5 times higher than with opioid use alone. Then, for those who used both medications for longer than 90 days and did not overdose, the risk of overdose on days 91 to 180 of concurrent use was still almost double the risk of overdose with opioids alone. Patients who used opioids and benzodiazepines concurrently for more than 180 days without overdosing were not at an increased risk of overdose if they continued concurrent use beyond the 181st day compared with patients who only used opioids. However, these results do not mean that patients exposed to concurrent opioid and benzodiazepine use longer had lower risk of overdose; in fact, the longer the duration of concurrent use, the higher the risk of overdose, because the increased risk of overdose predicted during each time window (1-90, 91-180, 181-270, and 271 days of concurrent use) would be cumulative. Although our observational analysis cannot provide a biological explanation for our findings, our results are biologically plausible from a pharmacological perspective. The higher risk of overdose observed in the first days of concurrent opioid and benzodiazepine use is consistent with the temporality commonly observed for the risk of adverse events related to medication interactions, which is higher in the first days with concurrent use of potentially interacting drugs. The decreased risk of overdose over time can be explained by the development of tolerance to the effect of central nervous system depressors.

Our findings have important implications from both the clinical and policy perspectives. First, despite the known increased risk of overdose associated with concurrent opioid and benzodiazepine use, it is very common in the Medicare population. This high prevalence is particularly concerning in light of our finding that the risk of overdose is highest during the first days of concurrent administration of benzodiazepines. Clinicians should avoid prescribing benzodiazepines to patients using opioids. When the concurrent use of both medications is medically necessary, patients should be closely monitored, particularly during the first days of concurrent use. Second, the design of policy interventions should evolve from the current objective of penalizing the long-term concurrent use of both medications to preventing their concurrent use in the first place. For instance, the Pharmacy Quality Alliance recently introduced a new performance measure defined as the proportion of patients from a health insurance carrier of a clinician who had 15 days or more with overlapping supplies of opioids and benzodiazepines.^17^ The application of this performance measure would disincentivize the prescription of benzodiazepines to patients using opioids for more than 15 days; however, it will not prevent concurrent use. Our results suggest that performance measures should be redefined to not only encourage insurance carriers and clinicians to limit the length of exposure to both medications, but to limit the number of patients who are exposed to both of them simultaneously at all. Third, when we performed sensitivity analyses and did not control for the number of opioid and benzodiazepine prescribers, our estimates for the HR of overdose with concurrent opioid and benzodiazepine use increased considerably. This demonstrates that the number of opioid and benzodiazepine prescribers is a strong confounder in estimating the association between concurrent opioid and benzodiazepine use and the risk of overdose and needs to be adjusted for. The association between number of opioid prescribers and risk of overdose was also described by prior studies.^8,9,10,11^ However, our findings are an important contribution to this existing literature because we showed that not only the number of opioid prescribers, but also the number of benzodiazepine prescribers, was associated with increased risk of overdose. Overall, these results demonstrate the important role that fragmentation of care plays in the inappropriate use of opioids and in the subsequent risk of overdose, and warrant the extended use of prescription monitoring programs and the implementation of new policy interventions that further control the receipt of opioid prescriptions by multiple prescribers. For example, states could require clinicians to check the prescription drug monitoring programs before prescribing benzodiazepines for patients with a history of opioid use, as it is currently done in some states for certain controlled substances.^18^

### Limitations

Our results are subject to several limitations. First, we used claims data, which only contain information about the prescriptions filled by patients and not about whether patients actually take the medications. In calculating the number of days with overlapping supplies of opioids and benzodiazepines, we assumed that patients took their medications as directed. However, it is possible that patients who possessed both opioids and benzodiazepines on a given day did not take one of them. In addition, claims data do not contain information on prescriptions filled without going through insurance. As a result, our analyses did not capture opioid prescriptions paid with cash, and we could not control for the use of antihistamine agents, which are also central nervous system depressants but are often purchased over the counter. Second, in our analyses, we did not stratify or control by the type of benzodiazepine use; however, this should not be concerning because prior analyses have shown that estimates for the increased risk of overdose associated with concurrent opioid and benzodiazepine use are consistent across benzodiazepines.^7^ Third, our study sample included beneficiaries who may have resided in nursing homes at some points during the study. Patterns of opioid prescribing may be different in the nursing home than in the outpatient setting; future studies should conduct similar analyses stratifying by nursing home admission. Finally, because our claims data only captured Medicare beneficiaries, our results may not be generalizable to the entire US population, particularly to younger patients.

## Conclusions

In this retrospective study of Medicare Part D claims data from 2013 to 2014, we found that the first 90 days with concurrent benzodiazepine use are associated with a 5-fold increase in the risk of overdose associated with opioids. Higher number of opioid and benzodiazepine prescribers, a proxy for care fragmentation, was associated with increased risk of overdose and was a strong confounder in examining the association between concurrent use of opioids and benzodiazepines and the risk of overdose. In those cases where the concurrent administration of benzodiazepines is medically necessary, patients should be closely monitored, particularly during the first days of concurrent use. The development and implementation of policy interventions that encourage insurance carriers and clinicians to limit not only the length of exposure to both medications, but also the number of patients who are simultaneously exposed to both of them in the first place, is warranted.

## Notes

Supplement. **eFigure.** Kaplan-Meier Curves for Opioid-Related Overdose
