# Supplementary material for: Exposure-Response Association Between Concurrent Opioid and Benzodiazepine Use and Risk of Opioid-Related Overdose in Medicare Part D Beneficiaries
Source: JAMA Netw Open. 2018 Jun 22;1(2):e180919. doi: 10.1001/jamanetworkopen.2018.0919 (PMC6324417; doi:10.1001/jamanetworkopen.2018.0919)
Supplement: Supplement. — eFigure. Kaplan-Meier Curves for Opioid-Related Overdose [file jamanetwopen-1-e180919-s001.pdf]

Supplementary Online Content

Hernandez I, He M, Brooks MM, Zhang Y. Exposure-response association between concurrent opioid and benzodiazepine use and risk of opioid-related overdose in Medicare Part D beneficiaries. *JAMA Netw Open*. 2018;1(2):e180919. doi:10.1001/jamanetworkopen.2018.0919

eFigure. Kaplan-Meier Curves for Opioid-Related Overdose

This supplementary material has been provided by the authors to give readers additional information about their work.

© 2018 Hernandez I et al. *JAMA Network Open.*

eFigure. Kaplan-Meier Curves for Opioid-Related Overdose.

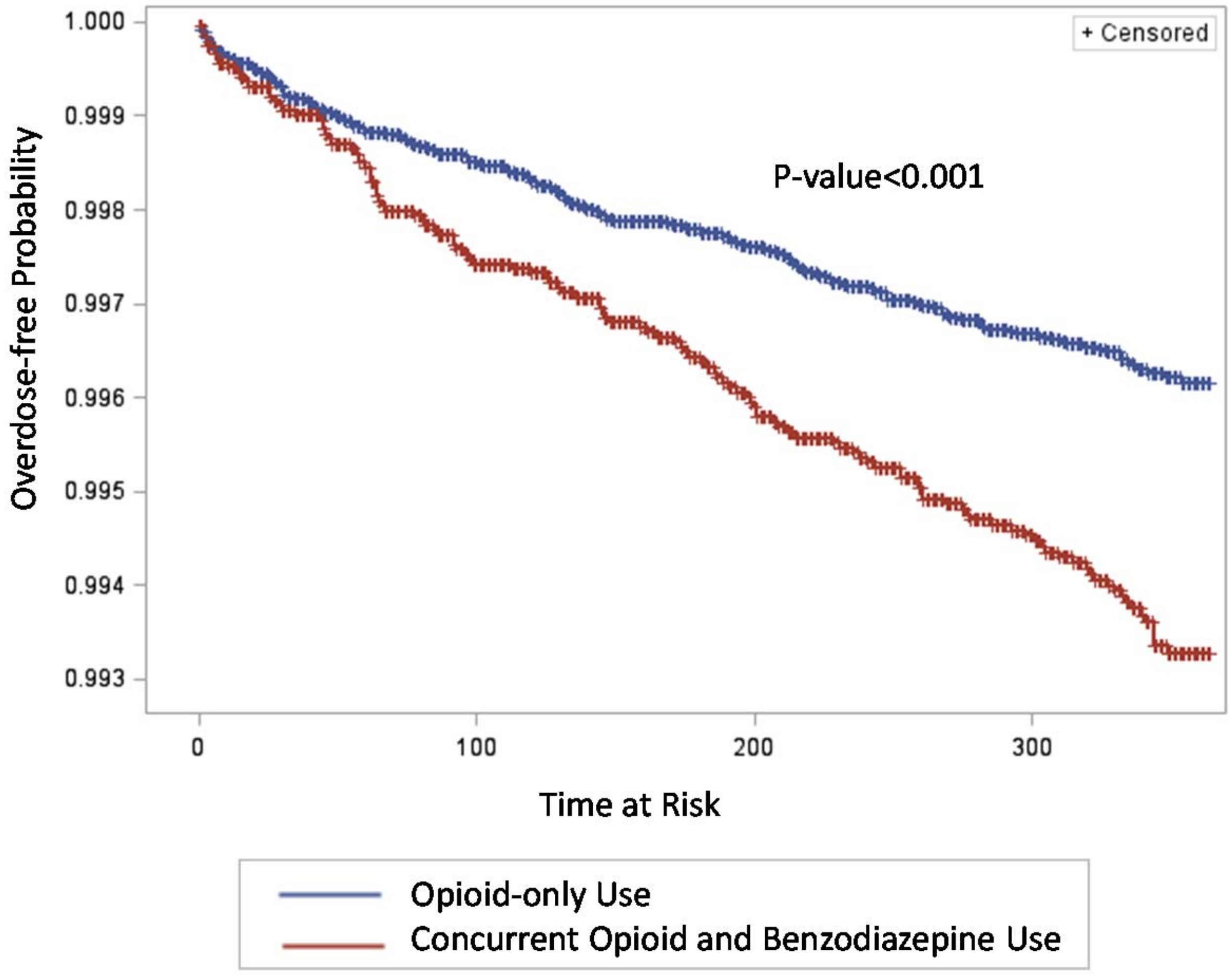

© 2018 Hernandez I et al. *JAMA Network Open.*
